# Radiological and functional evidence of the bronchial spread of tuberculosis: an observational analysis

**DOI:** 10.1016/S2666-5247(21)00058-6

**Published:** 2021-10

**Authors:** Ray Y Chen, Xiang Yu, Bronwyn Smith, Xin Liu, Jingcai Gao, Andreas H Diacon, Rodney Dawson, Michele Tameris, Hong Zhu, Yahong Qu, Ruanqing Zhang, Shouguo Pan, Xiaowei Jin, Lisa C Goldfeder, Ying Cai, Kriti Arora, Jing Wang, Joel Vincent, Stephanus T Malherbe, Friedrich Thienemann, Robert J Wilkinson, Gerhard Walzl, Clifton E Barry

**Affiliations:** a Tuberculosis Research Section, Laboratory of Clinical Immunology and Microbiology, Division of Intramural Research, National Institute of Allergy and Infectious Disease, National Institutes of Health, Bethesda, MD, USA; b Wellcome Centre for Infectious Diseases Research in Africa, University of Cape Town, Cape Town, South Africa; c Institute of Infectious Disease and Molecular Medicine, University of Cape Town, Cape Town, South Africa; d Division of Pulmonology, Department of Medicine, University of Cape Town Lung Institute, University of Cape Town, Cape Town, South Africa; e Department of Medicine, Faculty of Health Sciences, University of Cape Town, Cape Town, South Africa; f South African Tuberculosis Vaccine Initiative, University of Cape Town, Cape Town, South Africa; g DST-NRF Centre of Excellence for Biomedical Tuberculosis Research, South African Medical Research Council Centre for Tuberculosis Research, Division of Molecular Biology and Human Genetics, Department of Biomedical Sciences, Faculty of Medicine and Health Sciences, Stellenbosch University, Cape Town, South Africa; h Department of Medicine, Stellenbosch University, Cape Town, South Africa; i Henan Provincial Chest Hospital, Zhengzhou, Henan, China; j Sino-US Tuberculosis Collaborative Research Program, Zhengzhou, Henan, China; k TASK Applied Science, Cape Town, South Africa; l Kaifeng City Institute of Tuberculosis Prevention and Control, Kaifeng, Henan, China; m Xinxiang City Institute of Tuberculosis Prevention and Control, Xinxiang, Henan, China; n Zhongmu County Health and Epidemic Prevention Station, Zhongmu, Henan, China; o Xinmi City Institute of Tuberculosis Prevention and Control, Xinmi, Henan, China; p Clinical Monitoring Research Program Directorate, Frederick National Laboratory for Cancer Research, Frederick, MD, USA; q Department of Internal Medicine, University Hospital Zurich, University of Zurich, Zurich, Switzerland; r Francis Crick Institute, London, UK; s Department of Infectious Diseases, Imperial College London, London, UK

## Abstract

**Background:**

Direct bronchial spread of tuberculosis was extensively described in pre-antibiotic human pathology literature but this description has been overlooked in the post-antibiotic era, in which most pathology data come from animal models that emphasise the granuloma. Modern techniques, such as [^18^F]2-fluoro-2-deoxy-D-glucose (FDG) PET-CT scans, might provide further insight. Our aim was to understand normal early tuberculosis resolution patterns on pulmonary PET-CT scans in treated patients with tuberculosis who were subsequently cured.

**Methods:**

In this observational analysis, we analysed data from PredictTB, an ongoing, prospective, randomised clinical trial that examined sequential baseline and week 4 FDG-PET-CT scans from participants successfully treated (sputum culture negative 18 months after enrolment) for drug-susceptible pulmonary tuberculosis in South Africa and China. Participants who were aged 18–75 years, GeneXpert MTB/RIF positive for tuberculosis and negative for rifampicin resistance, had not yet started tuberculosis treatment, had not been treated for active tuberculosis within the previous 3 years, and met basic safety laboratory criteria were included and participants with diabetes, HIV infection, or with extrapulmonary tuberculosis including pleural tuberculosis were excluded. Scans were assessed by two readers for the location of tuberculosis lesions (eg, cavities and consolidations), bronchial thickening patterns, and changes from baseline to week 4 of treatment.

**Findings:**

Among the first 124 participants (enrolled from June 22, 2017, to Sept 27, 2018) who were successfully treated, 161 primarily apical cavitary lesions were identified at baseline. Bronchial thickening and inflammation linking non-cavitary consolidative lesions to cavities were observed in 121 (98%) of 124 participants' baseline PET-CT scans. After 4 weeks of treatment, 21 (17%) of 124 participants had new or expanding lesions linked to cavities via bronchial inflammation that were not present at baseline, particularly participants with two or more cavities at baseline and participants from South Africa.

**Interpretation:**

In participants with pulmonary tuberculosis who were subsequently cured, the location of cavitary and non-cavitary lesions at baseline and new lesions at week 4 of treatment suggest a cavitary origin of disease and bronchial spread through the lungs. Bronchial spread from cavities might play a larger role in the spread of pulmonary tuberculosis than has been appreciated. Elucidating cavity lesion dynamics and *Mycobacterium tuberculosis* viability within cavities might better explain treatment outcomes and why some patients are cured and others relapse.

**Funding:**

Bill & Melinda Gates Foundation, European and Developing Countries Clinical Trials Partnership, China Ministry of Science and Technology, National Natural Science Foundation of China, and National Institutes of Health.

**Translations:**

For the Chinese, Afrikaans and Xhosa translations of the abstract see Supplementary Materials section.

## Introduction

Tuberculosis infection has traditionally been described in two stages, latent or active. In the past 5 years, reviews of tuberculosis, however, have begun to view infection more as a continuum rather than distinct, dichotomous stages. Driven by data from rabbits and non-human primates, it is thought that *Mycobacterium tuberculosis* bacilli contained in granulomas begin to replicate and erode into a bronchus, forming a cavity that then allows transmission until symptoms emerge and a patient is treated.^1^, ^2^ However, pathological descriptions of autopsy specimens that were commonly done during the pre-antibiotic era, up to the 1950s, from patients who died of tuberculosis identified a more complex natural history of disease progression. Pathologists of that time recognised that primary tuberculosis infection generally did not lead to active tuberculosis but rather was most often controlled within a granuloma or the hilar lymph nodes. The resumption of *M tuberculosis* bacillary replication followed by spread within the lungs eventually led to active tuberculosis and its spread. Multiple authors noted that cavities formed from a caseous focus of infection that progressed to liquefactive necrosis. When such a lesion expanded and eroded into a bronchus, air entered the lesion to form a cavity and the liquefied lesion contents were released through the bronchus to infect other parts of the lungs, causing bronchogenic consolidations.^3^, ^4^, ^5^, ^6^, ^7^, ^8^, ^9^, ^10^ These observations from over 60 years ago provide key insights into how active tuberculosis often originates in lesions that become cavities, with bronchial spread of liquefied cavity contents causing disease elsewhere in the lungs and passing out through the mouth as phlegm or sputum. The pathologists of the time thus recognised that cavity appearance and formation was inextricably linked to bronchial spread within the patient and to subsequent transmission.^3^, ^7^, ^8^, ^9^, ^11^, ^12^

**Research in context**

**Evidence before this study**

Many radiology and pathology studies in patients with tuberculosis have been done previously. We searched PubMed for the terms “bronchial spread” or “bronchogenic spread” and “tuberculosis” from database inception to Dec 15, 2020, with no language restrictions. 89 articles were identified. These articles and their references were searched, as well as three available tuberculosis textbooks from the 1940s and 1950s. In studies of CT scans of patients with pulmonary tuberculosis, bronchogenic spread is described as nodular lesions or with the tree-in-bud sign. However, multiple pathology studies done before 1950 before the advent of tuberculosis chemotherapy suggested that bronchial spread of tuberculosis originated from cavities and caused much more severe disease including consolidations. With the arrival of effective tuberculosis chemotherapy, autopsies of patients with tuberculosis were generally no longer done. Animal models of tuberculosis were used instead but these models did not effectively reflect the reactivation tuberculosis that is most commonly seen in human adults with tuberculosis, leading to a shift in focus to the tuberculosis granuloma rather than the cavity.

**Added value of this study**

Historical pathological autopsy descriptions of human pulmonary tuberculosis have never been correlated with modern radiological studies to inform our understanding of the pulmonary tuberculosis pathology seen radiologically. Before starting treatment, 121 (98%) of 124 participants in our study who were eventually cured of their pulmonary tuberculosis showed patterns on PET-CT scans consistent with bronchial spread of tuberculosis, primarily from cavities but occasionally from consolidations. This finding supports the older pathology literature. 21 (17%) of 124 particiants showed apparently worsening bronchial spread patterns (new or expanding lesions) 4 weeks after starting treatment, despite being subsequently cured.

**Implications of all the available evidence**

Cavities might spread tuberculosis disease to other parts of the lungs through the bronchi. Elucidating cavity lesion dynamics, including *Mycobacterium tuberculosis* viability within cavities, might be critical to better understanding treatment outcomes.

With the advent of effective chemotherapy starting in the 1950s, tuberculosis mortality dropped substantially such that, by the 1970s, mortality rates were so low that studies based on autopsy specimens were no longer practical.^13^ Pathology studies thereafter relied primarily on animal models, particularly rabbits, guinea pigs, and non-human primates, which develop extensive pathology. These models focused attention on the granuloma, a hallmark of primary tuberculosis infection, rather than on caseous pneumonias with cavitation and bronchial spread, seen in reactivation tuberculosis.^14^, ^15^, ^16^, ^17^, ^18^ There are few human pathology specimens available today to corroborate or refute the earlier studies, but data other than pathology might be informative. One analysis using [^18^F]2-fluoro-2-deoxy-D-glucose (FDG) PET-CT scans showed that new or worsening lesions appeared on the end-of-treatment month 6 scan in 34 (34%) of 99 participants treated in South Africa,^19^ suggesting that *M tuberculosis* might still be viable and possibly spreading among apparently cured patients. Although all eight participants with treatment failure had this mixed response pattern, so did 21 (28%) of the 76 cured participants, showing the poor prognostic specificity of this pattern. A better understanding of the pathophysiology behind these response patterns on PET-CT scans is needed to know how to interpret such new and seemingly worsening lesions that appear during treatment. To understand what treatment response patterns are associated with poor outcomes, we first need to understand the temporal range of patterns seen in eventually cured patients: tuberculosis pathology patterns on the baseline scan and how those patterns change during treatment. We are doing a tuberculosis clinical trial during which FDG-PET-CT scans are done at baseline and during treatment of patients with active tuberculosis. The aim of this analysis is to understand baseline and early tuberculosis treatment response patterns on pulmonary PET-CT scans in patients who are subsequently cured.

## Methods

### Study design and participants

The ongoing, prospective, randomised clinical trial Using Biomarkers to Predict TB Treatment Duration (PredictTB, NCT02821832) is a tuberculosis treatment-shortening study in participants with pulmonary, drug-sensitive tuberculosis with serial FDG-PET with high resolution non-contrast chest CT scans done at baseline, week 4 of treatment, and then at either week 16 or 24. Participants were enrolled in the Western Cape, South Africa, and in Henan Province, China, and were followed up until week 72 for final treatment outcomes. Full details of the study design, enrolment sites, inclusion and exclusion criteria, randomisation schema, statistical methods, and how PET-CT scans were done have been previously published.^20^ The study included patients aged 18–75 years (upper limit increased from 65 years during the trial to increase the enrolment rate), who were GeneXpert MTB/RIF positive for tuberculosis and negative for rifampicin resistance, had not yet started tuberculosis treatment, had not been treated for active tuberculosis within the previous 3 years, and met basic safety laboratory criteria. Participants with diabetes, HIV infection, or with extrapulmonary tuberculosis including pleural tuberculosis were excluded. Baseline PET-CT scans were done within 7 days of participant enrolment with the second scan done 4 weeks later (with a minus 3-day to plus 7-day window). Signed informed consent was obtained from each participant. This study was approved by the institutional review board (IRB) of the US National Institute of Allergy and Infectious Diseases (NIAID) and the IRB or ethics committee of each enrolling site. The first 124 participants cured in the trial (defined as sputum cultures that remained negative up until week 72), without regard to study arm assignment, were included in this observational analysis. This analysis is not related to the primary objective of the parent clinical trial.

### Procedures and statistical analysis

PET-CT scans were analysed using MIM version 6.7 (MIM Software, Cleveland, OH, USA) and Amira version 6.5 (Thermo Fisher Scientific, Waltham, MA, USA). Baseline characteristics between groups were analysed by *t* test for continuous variables and χ^2^ test or Fisher's exact test for categorical variables, with significance defined as p<0·05. Variables significant in the univariate model were tested together in a multivariate logistic regression model. A bronchial spread pattern of tuberculosis was defined as direct connections through inflamed or thickened bronchi between cavities, typically located in apical pulmonary segments of the upper or lower lobes, and consolidations, typically in dependent locations inferior to the cavities. Two readers (RYC and CEB III) independently assessed the segmental location of cavities and connected lesions, with inconsistencies resolved by consensus. Cavity volumes within individual segments were summed. The likelihood of bronchial spread to an inferior versus superior lung segment was tested by the Clopper-Pearson binomial test. R version 4.0.2 was used for the statistical analyses.

Sputum was collected from study participants at every follow-up visit and cultured on Löwenstein-Jensen solid medium and BACTEC mycobacterial growth indicator tube (Becton Dickinson) liquid medium.

### Role of the funding source

The Intramural Research Program of the NIAID, US National Institutes of Health, was involved in study design, data collection, data analysis, data interpretation, the writing of the report, and in the decision to submit the paper for publication. No other funding sources were involved in any of these decisions.

## Results

124 participants were enrolled from June 22, 2017, to Sept 27, 2018, and completed the study as cured (remained sputum culture negative) 18 months later (by April, 2020), the baseline characteristics are shown in the table. Among these participants, 161 cavities were seen (appendix 4 p 7). 115 (71%) of these 161 cavities were located in the apical S1 and posterior S2 segments of the right and left upper lobes, with an additional 36 (22%) in the apical S6 segment of the lower lobes. More than 90% of cavities were located in these apical segments, consistent with the known predilection of cavities in reactivation tuberculosis.^21^ Cavitary air volumes ranged widely from less than 1·0 mL to 104·5 mL (cavity volumes within individual segments were summed).

**Table:** Baseline characteristics of the 124 participants included in the study

		**Total (n=124)**	**No new or expanded lesion at week 4 (n=103)**	**New or expanded lesion at week 4 (n=21)**	p value*
Sex		..	..	..	0·61
	Female	37 (30%)	32 (31%)	5 (24%)	..
	Male	87 (70%)	71 (69%)	16 (76%)	..
Age, years		34·3 (18·6–69·2)	33·6 (18·6–69·2)	37·8 (21·0–62·0)	0·11
Weight, kg		56·9 (10·4)	56·8 (10·5)	57·6 (10·0)	0·73
Body-mass index		20·2 (3·2)	20·2 (3·2)	20·3 (3·2)	0·90
Current smoker†		59/86 (69%)	48/72 (67%)	11/14 (79%)	0·57
Previous smoker (not current smokers but report a history of smoking)†		27/86 (31%)	24/72 (33%)	3/14 (21%)	0·53
Duration of smoking, years		15·7 (0·7–42·0)	15·5 (0·7–42·0)	15·6 (1·0–30·0)	0·97
Cavities		..	..	..	0·0050
	0	9 (7%)	9 (9%)	0	..
	1	76 (61%)	68 (66%)	8 (38%)	..
	≥2	39 (31%)	26 (25%)	13 (62%)	..
Total cavity volume, mL		14·9 (20·3)	13·4 (19·2)	22·4 (24·2)	0·12
Country, n		..	..	..	0·034
	South Africa	87 (70%)	68 (66%)	19 (90%)	..
	China	37 (30%)	35 (34%)	2 (10%)	..

Data are n (%), mean (range), mean (SD), or n/N (%).

* *t* test for continuous variables and χ^2^ or Fisher's exact test for categorical variables.

† Missing values are because some participants did not declare smoking status.

**Figure 1:**
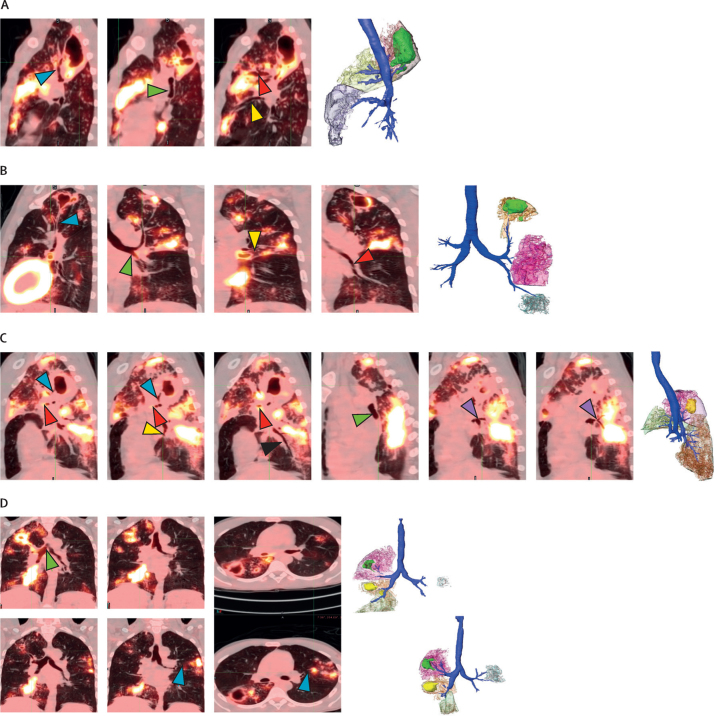
Examples of tuberculosis bronchial spread patterns on PET-CT scans with 3D renderings Images are of baseline scans unless otherwise noted. (A) Right upper lobe cavity drains down the S2 segment bronchus (blue arrow), across to the S3 segment bronchus (red arrow), and down the right bronchus intermedius (green arrow) to the S4 segment bronchus (yellow arrow), with spread of disease to those segments. The S2 segment lesion is red with a green cavity, S3 segment is yellow, and S4–5 segments are purple. (B) Bronchus (blue arrow) drains left S1–2 segment cavity straight down towards the left main bronchus (green arrow), branching to the S3 segment bronchus (yellow arrow) and down into the lower lobe (red arrow) to cause disease distal to those bronchi. In the 3D rendering, the S1–2 segment is orange with a green cavity, S3 segment is pink, and lower lobe disease is blue. (C) A bronchus (blue arrow) is visualised exiting the right S2 segment cavity, connecting down towards the right main bronchus (green arrow), with the S3 segment bronchus (red arrow) shooting off to cause disease anteriorly. S6 segment bronchus (purple arrow) comes off the right lower lobe bronchus and branches into the S6 segment (purple arrow) leading to disease in the apex of the right lower lobe. S7 bronchus (yellow arrow) and S10 bronchus (black arrow) are clearly outlined, leading to disease in those respective segments. In the 3D rendering, the S2 segment is pink with yellow cavity, the S3 segment is green, and the S6 and S10 segments are orange. (D) At baseline (top row), there are two cavities in the right upper lobe and another in the apical S6 segment of the right lower lobe. One or more of these spread tuberculosis bronchially down the right main bronchus (green arrow) to cause disease in the right lower lobe S7 and S8 segments. The left upper lobe is minimally involved at baseline with new or expanding S3 lesions appearing at week 4 (bottom row). At baseline, the right main bronchus inflammation from the right sided cavities spreads to the carina (green arrow). At week 4, the left main bronchus connects to the left S3 segment bronchi (blue arrows) and leads directly into the new or expanding lesion. The left S3 segment bronchus leading to the lesion was not well visualised at baseline, suggesting they became more inflamed by week 4, possibly from the increased bronchial spread. Bronchial spread across the carina like this could occur in a patient who sleeps on their left side at night. 3D=three-dimensional.

On baseline PET-CT scans of the 124 participants, nodular or consolidative lesions directly connected by thickened or inflamed bronchi to cavities were seen in 121 (98%) instances. Representative participants are shown in figure 1. The patient in figure 1A has a multi-lobed cavity in the right upper lobe segment S2. Thickened and inflamed bronchi can be traced from this cavity to consolidated lesions in dependent areas of the lungs in the right upper lobe S3 segment and the right middle lobe S4 and S5 segments, showing potential gravitational spread of disease through the bronchi. Figure 1B shows a patient with thickened bronchi connecting an apical left S1–2 cavity with a non-cavitary lesion in S3 and an inflamed left lower lobe bronchus leading to an area of consolidation in the left lower lobe. Figure 1C shows a patient with a large posterior right upper lobe S2 segment cavity leading to large consolidations in the right upper lobe anterior S3 segment and the apical S6 segment of the right lower lobe. In figure 1D, an inflamed right main bronchus at baseline connects two cavities in the right upper lobe with a large consolidation in the right lower lobe S7–8. A cavity in the apical right lower lobe S6 segment probably also contributed to the bronchial spread pattern in right lower lobe S7–8. There also appears to be bronchial spread of disease from the right side to the left upper lobe S3 segment at baseline but with increased spread noted at week 4 of treatment. Despite this apparent worsening of disease on treatment, this patient was subsequently cured. Additional examples of bronchial spread patterns are shown in appendix 4 (pp 8–11). In 32 (26%) of the 124 participants, the bronchial spread pattern appeared to originate in a consolidated lesion without a visualised cavity or with a very small (<1 mL) cavity (appendix 4 p 10).

**Figure 2:**
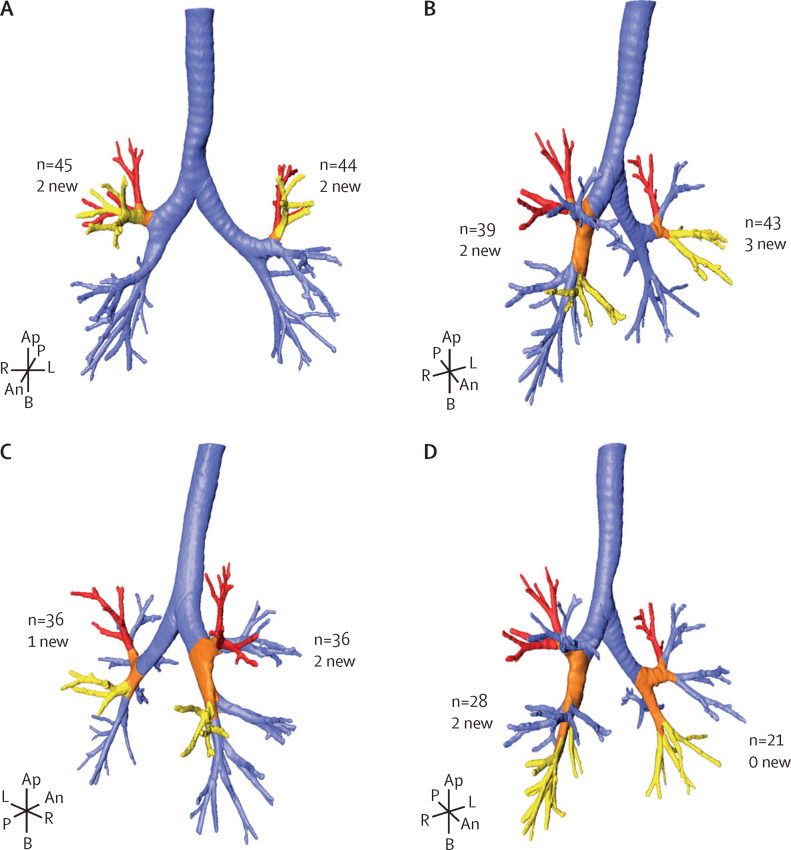
Ipsilateral bronchial spread pathways for apical segments 3D renderings of the source (cavitary) pulmonary segment (red), the bronchial spread pathway (orange), and the destination pulmonary segment (yellow) of selected bronchial spread pathways. The numbers indicate the total occurrences (baseline and week 4) of this pattern among the 124 participants analysed, with the number of new week 4 occurrences (subset of total). (A) Right lung segments S1–2 to right S3 and left lung segments S1–2 to left S3. (B) Right S1–2 to right S4–5 and left S1–2 to left S4–5. (C) Right S1–2 to right S6 and left S1–2 to left S6. (D) Right S1–2 to right S7–10 and left S1–2 to left S7–10. An=anterior. Ap=apical. B=basilar. L=left. P=posterior. R=right.

Across all 124 participants, we identified 490 non-cavitary consolidations connected to 161 cavitary lesions by thickened or inflamed bronchi. The most common pattern of apparent bronchial spread was from the apical-posterior S1–2 segments to the anterior S3 segment in both left and right lung lobes (figure 2A). The second most common pattern of bronchial spread was from S1–2 to S4 and S5, the right middle lobe and left lingula shown in figure 2B. Spread from S1–2 to the apical segment of the lower lung, S6 was also frequently observed (figure 2C). Spread to the lower lobe from S1–2 was less common but still seen (figure 2D). Additional bronchial spread patterns are shown in appendix 4 (pp 12–13). Of the total 161 cavities identified, the 151 (94%) apical segment (S1–2 and S6) cavities and the bronchial segments to which they spread their disease are described in appendix 4 (pp 2–3). The remaining ten (6%) cavities are described in appendix 4 (pp 4–5). Disease spread predominantly to the ipsilateral lung but spread to the contralateral upper lobe was also seen (appendix 4 pp 2–3). To provide further evidence that inferior, dependent spread was more likely than superior spread, we analysed the likelihood of spread from an S6 segment cavity to inferior segments (ipsilateral S4–10) or superior segments (ipsilateral S1–3 or any contralateral segment). We chose S6 segment cavities to model because S1–2 segment cavities can only spread inferiorly. Spread of lesions was significantly more likely to inferior, dependent segments of the lung (55 [64%] of 86), than to superior segments (31 [36%] of 86, p=0·013).

**Figure 3:**
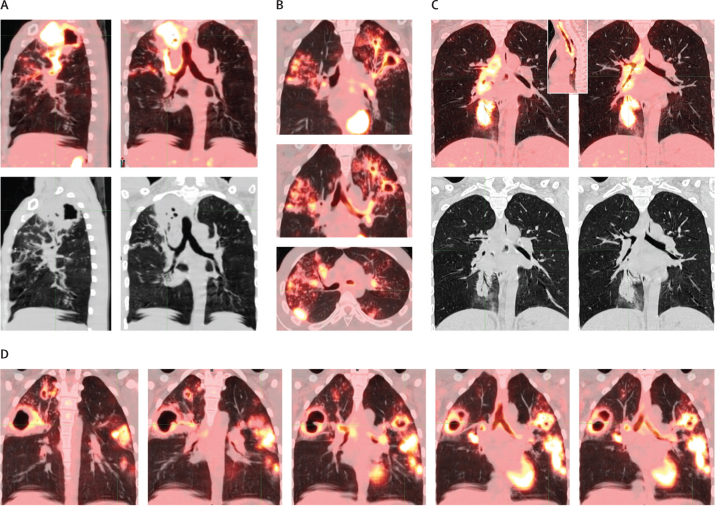
Examples of bronchial inflammation on baseline PET-CT scan (A) Inflammation from right S2 segment cavity flows down bronchus, which is better seen on CT alone (black and white image), into the right main bronchus and trachea. (B) Inflammation from left upper lobe cavities flows down into the left main bronchus. Although inflammation is not visualised crossing the carina, there is a direct bronchial connection to right S2 and S3 segment lesions. (C) Right lower lobe S7 segment consolidation with inflammation travelling up the right main bronchus and trachea to the pharynx (inset image shows the the trachea to pharynx; black and white image is CT alone). (D) Right and left S3 segment cavities with bronchial inflammation connecting the two across the carina, spreading inferiorly to the left S6 segment. Figures A–C are from the PredictTB study. Figure D is from a patient in a different study (NexGen EBA: Radiologic and Immunologic Biomarkers of Sterilizing Drug Activity in Tuberculosis, NCT02371681).

Intense FDG uptake (standardised uptake value >3) was observed in the bronchial lumen of ten (8%) of 124 participants. This finding could represent bronchial spread of liquefied cavitary contents or its associated inflammation, or both. Figure 3 shows four examples of clearly inflamed bronchi connecting cavities with other consolidations, travelling up the trachea, and even crossing the carina. Bronchial spread across the carina could be dependent spread in a person who tends to sleep on their side at night, and has been previously described from autopsy specimens.^7^ On occasion, bronchial spread patterns appear to travel from an inferior cavity to a superior segment, which could possibly occur via aspiration of bacilli backwards (described historically^9^), or with coughing, with liquefied material travelling superiorly in a bronchus other than the trachea.

**Figure 4:**
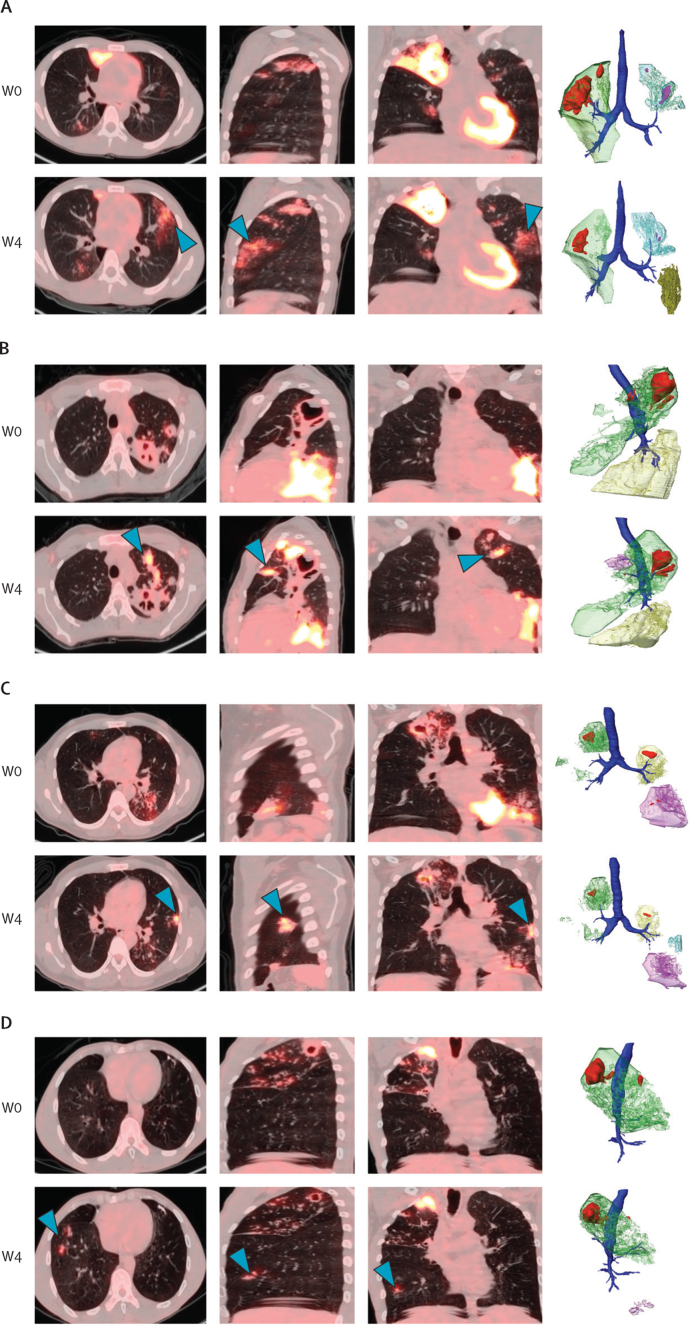
Representative examples of new lesions on the W4 PET-CT scan with 3D renderings (A) Large right upper lobe cavity (red) with surrounding dense consolidation (green) and smaller left segments 1–3 cavities (pink) with surrounding consolidation (blue). All cavities shrank by W4. There was a new left side lingular infiltrate (blue arrows, green-brown lesion on 3D rendering) at W4 that was not present at W0. (B) At baseline, there was a large posterior left upper lobe S1–2 cavity (red) with small anterior S3 cavity (red) at baseline and associated consolidation (green) primarily in left S1–2 down to the lingula S4–5. There was also an extensive left lower lobe consolidation (yellow) as well. At W4, left S1–2 segment cavity had decreased in size but with markedly increased PET uptake around it. Left lower lobe consolidation, however, had improved. There was a new left S3 segment consolidation (blue arrows, pink lesion on 3D rendering) that was not present at baseline. (C) At baseline, there were cavities (red) with associated consolidations in the right upper lobe, apex of the left lower lobe, and in the left base. At W4, all of these had improved but there was a new left lingular lesion (blue arrows, blue lesion on 3D rendering) that was not present at baseline. (D) At baseline, disease was confined to the right upper lobe with a S1 cavity, surrounding consolidation, and spread of disease to the S3 segment. At W4, there were two new foci of nodular disease (blue arrows, purple lesion on the 3D rendering) in the right lower lobe S8 segment. 3D=three-dimensional. W=week.

On the week 4 scans, 21 (17%) of 124 participants developed a total of 26 new or expanding lesions that were not present on their baseline scans with a direct bronchial connection to a superior cavity (appendix 4 pp 2–5). Among these 21 participants, those with new or expanding lesions were significantly more likely to have two or more cavities (compared with zero or one cavity, p=0·0046) and to be from South Africa (compared with China, p=0·034; table). Both cavity number and country remained significant with regard to developing a new or expanded lesion at week 4 in the final multivariate logistic regression model (cavity ≥2, odds ratio [OR] 4·79 [95% CI 1·78–13·70], p=0·0024; South Africa, 4·86 [1·25–32·40], p=0·045; appendix 4 p 6). An example of an expanding lesion is shown in figure 1D, in which the left upper lobe infiltrate present at baseline is expanded at week 4. Examples of new lesions that appear at week 4 are shown in figure 4. These new or expanding lesions originated from an apical S1–2 or S6 cavity except for one, which spread from a left upper lobe S3 segment cavity to the right upper lobe S3 segment (appendix 4 pp 4–5). 21 (81%) of 26 new or expanding lesions originated from an apical S1–2 cavity and seven (27%) resulted in a lesion in the contralateral, previously unaffected lung from the originating cavitary lesion and, therefore, must have traversed the carina. Despite this seeming worsening of disease, all these participants were subsequently cured. In a microbiological analysis of solid culture results (cultures grown on Löwenstein–Jensen medium), 19 (90%) of 21 participants with new or expanding week 4 lesions sputum culture converted to negative by week 8, compared with 98 (95%) of 103 participants without new or expanding lesions (log-rank p=0·18). Week 8 liquid culture conversion rates by mycobacterial growth indicator tube between participants with or without new or expanding week 4 lesions also did not differ (log-rank p=0·44).

## Discussion

This observational analysis of the baseline and week 4 PET-CT scans of the first 124 participants to complete the PredictTB trial and be cured of tuberculosis supports historical descriptions of reactivation tuberculosis, in which cavities are the source of bronchial spread of disease to consolidative lesions in dependent portions of the lungs. 98% of the participants in this cohort had bronchial thickening that characterises the bronchial spread patterns on baseline PET-CT scans. Only three participants with relatively mild disease did not have any bronchial thickening. The flow of inflammatory debris through the thickened bronchi is not visualised in most of the PET-CT scans but ten (8%) participants in this cohort did have clearly inflamed bronchi, evidenced by FDG PET uptake in addition to thickened bronchi, into other parts of the lung and even up the trachea, consistent with expulsion of liquefied phlegm through coughing (figure 2). Phlegm is filled with neutrophils,^22^ which take up FDG. Additional bronchial spread patterns with thickened bronchi persisted even 4 weeks into treatment in a minority (21 [17%]) of the 124 participants, appearing as new or expanded lesions (figure 1D, figure 4), which was more likely to occur in participants with two or more cavities at baseline (compared with ≤1 cavity) and among those from South Africa (compared with China).

Multiple radiological descriptions of tuberculosis have been published, with evidence for bronchogenic spread of tuberculosis seen on CT scans described as 2–4 mm centrilobular nodules and linear branching opacities around terminal bronchioles (tree-in-bud sign).^23^, ^24^ Although these findings might be evidence of local bronchial spread of tuberculosis, the distant spread to other ipsilateral or contralateral pulmonary segments from cavities via the bronchi has not, to our knowledge, been previously described radiologically, including descriptions of FDG PET uptake within the bronchi. Endobronchial tuberculosis has been well described and might affect more than half of patients with acute pulmonary tuberculosis. Endobronchial tuberculosis is primarily diagnosed on bronchoscopy and has a variable appearance, ranging from bronchial mucosal oedema or hyperaemia to ulcerative or mass lesion, or both, with the main complication being bronchial stenosis.^25^ It is probable that some of our participants had endobronchial tuberculosis but, as we did not do any bronchoscopies, we are unable to determine which participants were affected and the relationship of endobronchial tuberculosis with the bronchial spread patterns that we describe.

Bronchial spread patterns of tuberculosis are present in most participants at baseline, which suggests that bronchial spread occurs in the natural history of untreated pulmonary tuberculosis. At week 4 of treatment, however, new or expanding lesions were seen in 21 (17%) of 124 cured participants, particularly those with two or more cavities at baseline and those from South Africa. The finding that participants with more cavities had additional bronchial spread patterns is consistent with cavities being the source of these patterns. Why cavity number but not volume was associated with bronchial spread patterns is not clear and needs to be further explored. Additional analyses are also needed to understand why South African participants had more bronchial spread patterns than Chinese participants. Some of these new or expanding lesions could be subclinical paradoxical reactions to treatment rather than true spread of disease. A review of paradoxical tuberculosis reactions in patients without HIV found the prevalence to be between 2–23%.^26^ Alternatively, bronchial spread after the start of treatment might be a chemical pneumonitis reaction to released bacterial antigen than actual disease spread, which has also been described historically,^7^ and might explain why bronchial spread patterns at week 4, and possibly even at month 6,^19^ might not indicate a worse prognosis. Sputum culture conversion results being similar between patients with and without new or expanding week 4 lesions support this explanation. More definitive analyses of how bronchial spread patterns associate with final treatment outcomes, cavity number and volume, and country will require including participants with poor outcomes, which will be done at the end of the parent PredictTB trial.

We believe that the bronchial thickening seen on PET-CT scans is caused by the flow of cellular debris and possibly infected neutrophils resulting from the host's inflammatory response to necrotic lesions that connect to a bronchus and become cavities. One limitation to this conclusion, however, is that we do not have histological evidence of bronchial spread, and that what is being shown on the PET scans is inflammation of blood or lymphatic vessel walls, which travel along the bronchi. Although *M tuberculosis* bacilli are known to spread via the vasculature or lymphatics, the bronchial spread patterns we see, predominantly dependent spread to different ipsilateral or contralateral lung lobes, are not consistent with vascular or lymphatic seeding. Haematogenous dissemination, if slight, might lead to scattered foci of infection in the lungs, whereas heavy dissemination would lead to miliary tuberculosis. Neither type of dissemination should have a distribution defined by bronchial anatomy with primarily dependent spread. Lymphatic spread would seed bacilli proximally, typically infecting the hilar lymph nodes. Seeding ipsilateral lung lobes or crossing the carina to the contralateral lung would require first proximal then distal movement of bacilli via the lymphatics, which would be unlikely. Such movement, however, is consistent with direct, dependent bronchial spread of necrotic material containing *M tuberculosis* bacilli, for which there is abundant historical histological support. Another limitation of our study is that with static radiology images, connections between lesions can be seen but the directionality of the spread, or even whether there is any spread at all, cannot be definitely concluded. However, cavities on baseline chest x-ray have long been associated with poor treatment outcomes in multiple studies.^27^, ^28^, ^29^, ^30^ In our study, multiple baseline cavities (≥2) are also associated with a significantly increased odds of a new or expanding lesion at week 4. These associations, along with pathology data from before the 1950s, support the cavitary origin of bronchial spread. The historical descriptions also include cases of bronchial spread patterns originating in consolidations without a visualised cavity or with a very small (<1 mL) cavity,^8^, ^9^ which was seen in 26% of our participants.

The concept of bronchial spread from cavities in pulmonary tuberculosis that was extensively described in the pre-antibiotic era has been mostly lost in the intervening 60 years or so because of the scarcity of human autopsy specimens and subsequent animal models that do not completely recapitulate human disease.^31^ However, pulmonary PET-CT scans show changes consistent with bronchial spread of tuberculosis from cavities in almost all participants at baseline and continued bronchial spread patterns in a minority, particularly among those with two or more cavities, even 4 weeks after starting treatment. This finding suggests that cavities might spread tuberculosis disease to other parts of the lungs through the bronchi and specifically targeting treatment to cavities might be more important than previously realised. Not all drugs penetrate and sterilise cavities well^32^ so those that do might be more effective to cure disease and prevent relapse. A better understanding of cavitary lesion dynamics and the viability of *M tuberculosis* within cavities might provide an effective biomarker of cure. Additionally, new or expanded nodular or consolidative lesions that appear on radiology after starting treatment might not always indicate disease worsening or drug resistance. Additional studies with more participants, especially those with poor outcomes, are needed to support these findings. After the overall PredictTB study has concluded, analyses will be done to understand more fully these bronchial spread patterns and their associations with final treatment outcomes.

## Data sharing

Deidentified participant data will be made available by request to the corresponding author following the completion of the overall PredictTB clinical trial according to the data sharing policies of the funders.

## Declaration of interests

RJW is supported by Francis Crick Institute, which receives funding from UK Research and Innovation (FC0010218), Wellcome (FC0010218), and Cancer Research UK (FC0010218). RJW also receives support from Wellcome (104803, 203135), the European and Developing Countries Clinical Trials Partnership (EDCTP; SRIA2015-1065), and US National Institutes of Health (NIH; AI115940). GW receives funding from the South African Research Chair Initiative from the National Research Foundation (86535). STM receives grants from the South African Medical Research Council (grant number CDF1576). KA is employed by the Bill & Melinda Gates Medical Research Institute which is a grantee of the Bill & Melinda Gates Foundation but a separate legal entity. All other authors declare no competing interests.

## Supplementary Materials

Chinese translation of the abstract

Afrikaans translation of the abstract

Xhosa translation of the abstract
