# Supplementary material for: Radiological and functional evidence of the bronchial spread of tuberculosis: an observational analysis
Source: Lancet Microbe. 2021 Oct;2(10):e518–26. doi: 10.1016/S2666-5247(21)00058-6 (PMC8478663; doi:10.1016/S2666-5247(21)00058-6)
Supplement: Chinese translation of the abstract [file mmc1.pdf]

THE LANCET
Microbe

Supplementary appendix 1

This translation in Chinese was submitted by the authors and we reproduce it as supplied. It has not been peer reviewed. *The Lancet*'s editorial processes have only been applied to the original in English, which should serve as reference for this manuscript.

此简体中文译文由作者提交，我方按照提供的版本刊登。此译文并未经过同行审阅。医学期刊《柳叶刀》的编辑流程仅适用于英文原稿，英文原稿应作为此手稿的参考。

Supplement to: Chen RY, Yu X, Smith B, et al. Radiological and functional evidence of the bronchial spread of tuberculosis: an observational analysis. *Lancet Microbe* 2021; published online July 6. https://doi.org/10.1016/S2666-5247(21)00058-6

肺结核由支气管扩散的放射学和功能学证据： 一项观察性研究

摘要

背景：在抗生素普及前的人类病理学文献中广泛描述了结核病由支气管直接播散，但在抗生素普及之后，这种描述被忽略了，因为大多数病理数据来自强调肉芽肿的动物模型。现代技术，如[18F]-2-氟-2-脱氧葡萄糖（FDG）PET/CT 扫描，可能会提供进一步的见解。我们的目的是了解被治愈的肺结核患者在治疗早期肺 PET/CT 扫描的分辨模式。

方法：在这项观察性研究中，我们分析了来自 Predict TB 的数据，这是一项正在南非和中国进行的对药物敏感性肺结核的前瞻性随机临床试验。该试验检查了参加者中治疗成功案例（入组后 18 个月 痰液培养阴性）的基线和第 4 周 FDG-PET/CT 扫描影像。纳入者年龄在 18～75 岁之间，GeneXpert MTB/RIF 检测 MTB 阳性且利福平耐药阴性，尚未开始结核病治疗，在过去 3 年内未因活动性结核病接受过治疗，实验室血检符合基本安全标准，且排除糖尿病、HIV 感染，及肺外结核（包括胸膜结核）。扫描影像由两位医师评估肺结核病灶的位置（如空洞、实变等）、支气管壁增厚模式，以及从基线到治疗第 4 周的变化。

结果：在目前成功治愈的 124 名参加者（2017 年 6 月 22 日至 2018 年 9 月 27 日入组）中，基线检查时确定了 161 个位于肺叶上部的原生病灶。在这 124 名参加者的基线 PET-CT 扫描影像中，有 121 名（98%）呈现支气管壁增厚和联系非空洞性实变病灶与空洞的炎症。治疗 4 周后，124 名参加者中有 21 名（17%）出现了新的或扩大的病变，这些病变通过支气管炎症与空洞相连，而这些病变在基线检查时并不存在，尤其是基线检查时空洞≥2 个的参加者和来自南非的参加者。

解释：在随后治愈的肺结核参加者中，基线检查时空洞和非空洞性病变的位置以及治疗第 4 周的新病变表明疾病扩散起源于空洞并通过支气管播散。来自空腔的支气管播散在肺结核的扩散

中所起的作用可能比人们所认识到的更大。阐明空洞病变动态和空洞内结核分枝杆菌的生存能力可能有助于更好地解释治疗结果，以及为什么一些患者治愈而另一些患者复发。

资助单位：比尔和梅琳达·盖茨基金会、欧洲和发展中国家临床试验合作关系、中国科技部、中国国家自然科学基金委员会、美国国立卫生研究院。
