# Supplementary material for: Radiological and functional evidence of the bronchial spread of tuberculosis: an observational analysis
Source: Lancet Microbe. 2021 Oct;2(10):e518–26. doi: 10.1016/S2666-5247(21)00058-6 (PMC8478663; doi:10.1016/S2666-5247(21)00058-6)
Supplement: Afrikaans translation of the abstract [file mmc2.pdf]

THE LANCET
Microbe

Supplementary appendix 2

This translation in Afrikaans was submitted by the authors and we reproduce it as supplied. It has not been peer reviewed. *The Lancet*'s editorial processes have only been applied to the original in English, which should serve as reference for this manuscript.

Hierdie vertaling in Afrikaans is deur die outeurs voorgelê en ons gee dit weer soos dit aan ons verskaf is. Dit is nie eweknie-beoordeel nie. The Lancet se redaksionele prosesse is slegs op die oorspronklike Engelse teks toegepas, en laasgenoemde moet as bronverwysing vir hierdie manuskrip gebruik word.

Supplement to: Chen RY, Yu X, Smith B, et al. Radiological and functional evidence of the bronchial spread of tuberculosis: an observational analysis. *Lancet Microbe* 2021; published online July 6. https://doi.org/10.1016/S2666-5247(21)00058-6

Radiologiese en funksionele bewyse van die brongiale verspreiding van tuberkulose: ’n ontleding op grond van waarneming

Opsomming

Agtergrond Regstreekse brongiale verspreiding van tuberkulose is omvattend omskryf in preantibiotiese literatuur oor menslike patologie. Hierdie omskrywing is egter oor die hoof gesien in die postantibiotiese era, waarin die meeste patologiese data verkry word uit dieremodelle, waarin granulome beklemtoon word. Hedendaagse tegnieke soos [18F]2-fluoor-2-deoksie-D-glukose- (FDG) PET-CT skandeerbeelde mag moontlik verdere insig bied. Die oogmerk met hierdie waarneming was om normale resolusiepatrone van vroeë tuberkulose op pulmonêre PET-CT-skandeerbeelde te beskryf in pasiënte wie tuberkulose behandeling suksesvol voltooi het en uiteindelik genees is.

Metodes In hierdie waarnemingsontleding is data ontleed wat bekom is uit PredictTB – ’n voortgesette, prospektiewe, ewekansige kliniese proefneming wat die sekwensiële basislyn- en week 4- FDG-PET-CT-skanderings ondersoek het van studie deelnemers in Suid-Afrika en China wat sukselsvol vir middelvatbare pulmonêre tuberkulose behandel was (d.w.s. wie se sputumkultuur na 18 maande vanaf inskrywing negatief getoets het). Deelnemers tussen 18 en 75 jaar wie se GeneXpert-MTB/RIF-positief vir tuberkulose en negatief vir rifampisienweerstandigheid was, nog nie met tuberkulosebehandeling begin het nie, nie binne die voorafgaande drie jaar vir aktiewe tuberkulose behandel is nie en aan basiese veiligheidskriteria voldoen het, is ingesluit. Deelnemers met diabetes, MIV-infeksie of ekstrapulmonêre tuberkulose (ook pleurale tuberkulose) is uitgesluit. Skanderings is deur twee lesers geassesseer vir die opsporing van tuberkulose letsels (bv. kavitasie en konsolidasie), brongiale verdikkingspatrone en veranderings vanaf die basislyn tot die vierde week van behandeling.

Bevindings Onder die eerste 124 deelnemers (ingeskryf vanaf 22 Junie 2017 tot 27 September 2018) wat suksesvol behandel is, is 161 hoofsaaklik atipiese kavitasies op die basislyn aangeteken. Brongiale verdikking en inflammasie wat ’n verband tussen soliede letsels en kavitasie aantoon, is in 121 (98%) van die 124 deelnemers se PET-CT-skandeerbeelde op die basislyn waargeneem. Na 4 weke van behandeling het 21 (17%) van die 124 deelnemers nuwe of vergrootte letsels vertoon wat met kavitasie via brongiale inflammasie verband hou en wat nie op die basislyn aanwesig was nie; dit was veral die geval onder deelnemers wat uit Suid-Afrika kom en wat twee of meer kavitasies op die basislyn vertoon het.

Vertolking In deelnemers met pulmonêre tuberkulose wat uiteindelik genees is, dui die ligging van kavitasie- en soliede letsels op die basislyn en nuwe letsels teen die vierde week van behandeling dat die siekte in die kavitasie ontstaan en brongiaal deur die longe versprei. Brongiale verspreiding vanaf die kavitasie mag ’n groter rol in die verspreiding van pulmonêre tuberkulose vervul as wat tot dusver besef is. Deur groter lig op die dinamiek tussen kavitasieletsels en die lewensvatbaarheid van *Mycobacterium tuberculosis* binne kavitasies te werp, behoort ons ’n beter begrip te vorm van behandelingsuitkomste en hoekom party pasiënte genees word en ander terugval.

Befondsing Bill & Melinda Gates Foundation, European and Developing Countries Clinical Trials Partnership, die Chinese Ministerie vir Wetenskap en Tegnologie, die Nasionale Natuurwetenskapstigting van China, en die National Institutes of Health (VSA)
