# Supplementary material for: Radiological and functional evidence of the bronchial spread of tuberculosis: an observational analysis
Source: Lancet Microbe. 2021 Oct;2(10):e518–26. doi: 10.1016/S2666-5247(21)00058-6 (PMC8478663; doi:10.1016/S2666-5247(21)00058-6)
Supplement: Xhosa translation of the abstract [file mmc3.pdf]

THE LANCET
Microbe

Supplementary appendix 3

This translation in Xhosa was submitted by the authors and we reproduce it as supplied. It has not been peer reviewed. *The Lancet*'s editorial processes have only been applied to the original in English, which should serve as reference for this manuscript.

Le nguqulelo yesiXhosa iye yafakwa ngababhali yaye siyayikhupha kutsha njengoko inikelwe. Ayikahlolisiswa zezinye iingcali. Iinkqubo zohlelo zeThe Lancet ziye zenziwa kuphela kumbhalo wokuqala ongesiNgesi, yaye kuya kufuneka ukuba zisetyenziswe njengembekiselo yalo mbhalo-ngqangi.

Supplement to: Chen RY, Yu X, Smith B, et al. Radiological and functional evidence of the bronchial spread of tuberculosis: an observational analysis. *Lancet Microbe* 2021; published online July 6. https://doi.org/10.1016/S2666-5247(21)00058-6

Ubungqina beradiyoloji nobusebenzayo bokusasazeka kwesifo sephepha kwimibhobho ethumela umoya emiphungeni: uhlalutyo olusekelwe kwimeko ebiyakhelwe umkhanya

Isishwankathelo

Intsusa Ukusasazeka ngokuthe ngqo kwesifo sephepha kwimibhobho ethumela umoya emiphungeni kwachazwa ngokubanzi kuncwadi oluthetha ngezigulo zoluntu kwixesha langaphambi kokusetyenziswa kwee-antibhayotiki kodwa le ngcaciso iye ayasiwa so kwixesha lasemva kokubakho kwee-antibhayotiki, apho inkcazelo eninzi ephathelele izigulo ithetha kakhulu ngegranyuloma. Ubugcisa bale mihla, njengezikena ze-[1^{8}F]2-fluoro-2-deoxy-D-glucose (i-FDG) i-PET-CT, zingasikrobisa kokungakumbi Injogo yethu yayikukuqonda iindlela esibonakala ngazo ngokuqhelekileyo xa sisaqala isifo sephepha kwizi keni ze-PET-CT zemiphunga kwizigulana ebezinesifo sephepha eziye zanyangwa kamva.

Iinkqubo Kolu hlalutyo lusekelwe ekwakheni umkhanya, siye sahlalutya inkcazelo efumaneka kwi-PredictTB, ulingo lonyango oluqhubekayo, olunokuba luncedo kwixesha elizayo, olukhethwa ngesiquphe oluye lwahlolisisa izikena ezisisiseko ezilandelelanayo nezeveki yesi-4, i-FDG-PET-CT zabathathi-nxaxheba abaye banyangelwa ngokuphumelelayo (abanezikhohlela ezinegethivu kwiinyanga ezili-18 emva kokubhalisa) isifo sephepha semiphunga eMzantsi Afrika naseTshayina. Abathathi-nxaxheba ababeneminyaka eli-18–75 ubudala, abaphozithivu kwiGeneXpert MTB/RIF enyanga isifo sephepha nabanegethivu kukuxhathisa kwe -rifampicin, abakaluqalisi unyango lwesifo sephepha, abakanyangelwa isifo sephepha esikhoyo kwisithuba seminyaka emi-3 edluleyo, baza bakulungela isiseko sokhuseleko kwaselebhu baye baqukwa kwaye abathathi-nxaxheba abanesifo seswekile, abane-HIV, okanye abanesifo sephepha esingaphandle kwemiphunga kuquka isifo sephepha kwinwebu yemiphunga baye abaqukwa. Izikena ziye zavandlakanywa ngabalesi ababini ukuze kubonwe indawo enezilonda zesifo sephepha (umz. imingxunya, nokuqokelelana), ukudumba kwemibhobho ehambisa umoya emiphungeni notshintsho ukususela kwisiseko ukuya kwiveki ye-4 yonyango.

Okufunyanisiweyo Phakathi kwabathathi-nxaxheba bokuqala abali-124 (ababhalise ukususela ngoJuni 22, 2017, ukuya kutsho ngoSeptemba 27, 2018) banyangwe ngempumelelo, 161 yezilonda yemingxunya ye apical yabonwa kuqala kwakwi nqanaba lesiseko. Ukudumba kwemibhobho ehambisa umoya emiphungeni nokudumba okunxulumene nezilonda ezingenamingxuma ukuya kutsho kwezinemingxuma ziye zaphawulwa kubathathi-nxaxheba abali-121 (98%) kwabo bali-124 kwizi keni ezisisiseko ze-PET-CT. Emva kweeveki ezi-4 zonyango, abathathi-nxaxheba abangama-21 (17%) kwabali-124 baye banezilonda ezitsha okanye ezandayo ezinxulumene nemingxuma edibana nokudumba kwemibhobho ethumela umoya emiphungeni, zinto ezo ebezingekho ebudeni bolingo olusisiseko, ingakumbi abathathi-nxaxheba abanemingxuma emibini nangaphezulu ebudeni bolingo olusisiseko nabathathi-nxaxheba baseMzantsi Afrika.

Ingcaciso Kubathathi-nxaxheba abanesifo sephepha semiphunga abathi banyangeka kamva, iindawo ezinezilonda ezinemingxuma nezingenamingxuma kulingo olusisiseko nezilonda ezitsha kwiveki ye-4 zonyango zibonisa ukuba esi sifo siqale ngomngxuma saza sanwenwela kwimibhobho ehambisa umoya saza sagqiba yonke imiphunga. Ukunwenwela kwaso kwimibhobho ehambisa umoya ukusuka emingxumeni kunokuba nendima enkulu kwisifo sephepha semiphunga kunokuba bekusaziwa ngaphambili. Iinkcukacha ezicace gca ngobubanzi zomngxuma wesilonda nokusebenza kwe*Mycobacterium tuberculosis* kwimingxuma kunokuyicacisa bhetele imiphumo yonyango nesizathu sokuba ezinye izigulana zinyangeke zize ezinye zibuyele umva.

Inkxaso-mali i-Bill & Melinda Gates Foundation, Ubudlelane BeYuriphu Kunye Namazwe Asakhasayo Kulingo Lonyango, ISebe LaseTshayina Lenzululwazi Nobugcisa, iZiko Lesizwe LaseTshayina Lenzululwazi Yezemvelo, kunye Namaziko Esizwe

EzeMpilo.
