# Supplementary material for: Radiological and functional evidence of the bronchial spread of tuberculosis: an observational analysis
Source: Lancet Microbe. 2021 Oct;2(10):e518–26. doi: 10.1016/S2666-5247(21)00058-6 (PMC8478663; doi:10.1016/S2666-5247(21)00058-6)
Supplement: Supplementary appendix 4 [file mmc4.pdf]

THE LANCET
Microbe

Supplementary appendix 4

This appendix formed part of the original submission and has been peer reviewed. We post it as supplied by the authors.

Supplement to: Chen RY, Yu X, Smith B, et al. Radiological and functional evidence of the bronchial spread of tuberculosis: an observational analysis. *Lancet Microbe* 2021; published online July 6. https://doi.org/10.1016/S2666-5247(21)00058-6.

Radiologic and functional evidence of the bronchial spread of tuberculosis: an observational analysis

Appendix

Table of Contents

Supplemental Table 1 2
Supplemental Table 2 4
Supplemental Table 3 6
Supplemental Figure 1 7
Supplemental Figure 2 8
Supplemental Figure 3 13

Supplemental Table 1

Summary of cavities located in upper lobe apical segments S1-S2 and lower lobe apical segments S6 and the pulmonary segments to which they spread disease among the 124 participants in the study. N represents lesions, not participants.

	Right Lung		Left Lung		Total	
	Total at baseline or week 4 combined	New lesions at week 4*	Total at baseline or week 4 combined	New lesions at week 4*	Total at baseline or week 4 combined	New lesions at week 4*
Pulmonary S1-S2 segment cavities (N):	58		57		115	
Bronchial spread location by pulmonary segments (%):						
ipsilateral S3	45 (77.6%)	2	44 (77.2%)	2	89 (77.4%)	4
ipsilateral S4-S5	39 (67.2%)	2	43 (75.4%)	3	82 (71.3%)	5
ipsilateral S6	36 (62.1%)	1	36 (63.2%)	2	72 (62.6%)	3
ipsilateral S7-S10	28 (48.2%)	2	21 (36.8%)	0	49 (42.6%)	2
contralateral S1-S3	24 (41.4%)	2	20 (35.1%)	1	44 (38.3%)	3
contralateral S4-S5	12 (20.7%)	2	7 (12.3%)	0	19 (16.5%)	2
contralateral S6-S10	14 (24.1%)	1	9 (15.8%)	1	23 (20.0%)	2
Pulmonary S6 segment cavities (N):	20		16		36	
Bronchial spread location by pulmonary segments (%):						
ipsilateral S1-S3	8 (40.0%)	0	4 (25.0%)	0	12 (33.3%)	0
ipsilateral S4-S5	15 (75.0%)	1	7 (43.8%)	1	22 (61.1%)	2
ipsilateral S7-S10	19 (90.5%)	2	14 (87.5%)	0	33 (91.7%)	2

contralateral S1-S3	5 (25.0%)	0	3 (18.8%)	0	8 (22.2%)	0
contralateral S4-S5	2 (10.0%)	0	4 (25.0%)	0	6 (16.7%)	0
contralateral S6-S10	3 (15.0%)	0	2(12.5%)	0	5 (13.9%)	0

* New week 4 lesions are a subset of the total for that lung

Supplemental Table 2

Summary of cavities located in non-apical segments and the pulmonary segments to which they spread disease among the 124 participants in the study. N represents lesions, not participants.

	Right Lung		Left Lung		Total	
	Total at baseline or week 4 combined	New lesions at week 4*	Total at baseline or week 4 combined	New lesions at week 4*	Total at baseline or week 4 combined	New lesions at week 4*
Pulmonary S3 segment cavities (N):	3		4		7	
Bronchial spread location by pulmonary segments (%):						
ipsilateral S1-2	2 (66.7%)	0	3 (75%)	0	5 (71.4%)	0
ipsilateral S4-S5	1 (33.3%)	0	4 (100%)	0	5 (71.4%)	0
ipsilateral S6	0		3 (75%)	0	3 (42.9%)	0
ipsilateral S7-S10	1 (33.3%)	0	3 (75%)	0	4 (57.1%)	0
contralateral S1-S3	1 (33.3%)	0	1 (25%)	1	2 (28.6%)	1
contralateral S4-S5	1 (33.3%)	0	0		1 (14.3%)	0
contralateral S6-S10	1 (33.3%)	0	0		1 (14.3%)	0
Pulmonary S4-5 segment cavities (N):	1		0		1	
Bronchial spread location by pulmonary segments (%):						
ipsilateral S1-S3	0					
ipsilateral S7-S10	1 (100%)	0			1 (100%)	0
contralateral S1-S3	0					
contralateral S4-S5	0					

contralateral S6-S10	0				
Pulmonary S7-10 segment cavities (N):	0	2		2	
Bronchial spread location by pulmonary segments (%):					
ipsilateral S1-3		1 (50%)	0	1 (50%)	0
ipsilateral S4-S5		1 (50%)	0	1 (50%)	0
ipsilateral S6-S10		2 (100%)	0	2 (100%)	0
contralateral S1-S3		0			
contralateral S4-S5		0			
contralateral S6-S10		0			

* New week 4 lesions are a subset of the total for that lung

Supplemental Table 3

Final multivariate logistic regression model for factors associated with new or expanding lesions on the week 4 PET/CT scan.

**Characteristic**	**OR**	**95% CI**	**P-value**
**Baseline Cavity Count**			
<2	—	—	
≥2	4.79	1.78, 13.70	0.0024
**Country**			
China	—	—	
South Africa	4.86	1.25, 32.40	0.045

OR = Odds Ratio, CI = Confidence Interval

Supplemental Figure 1

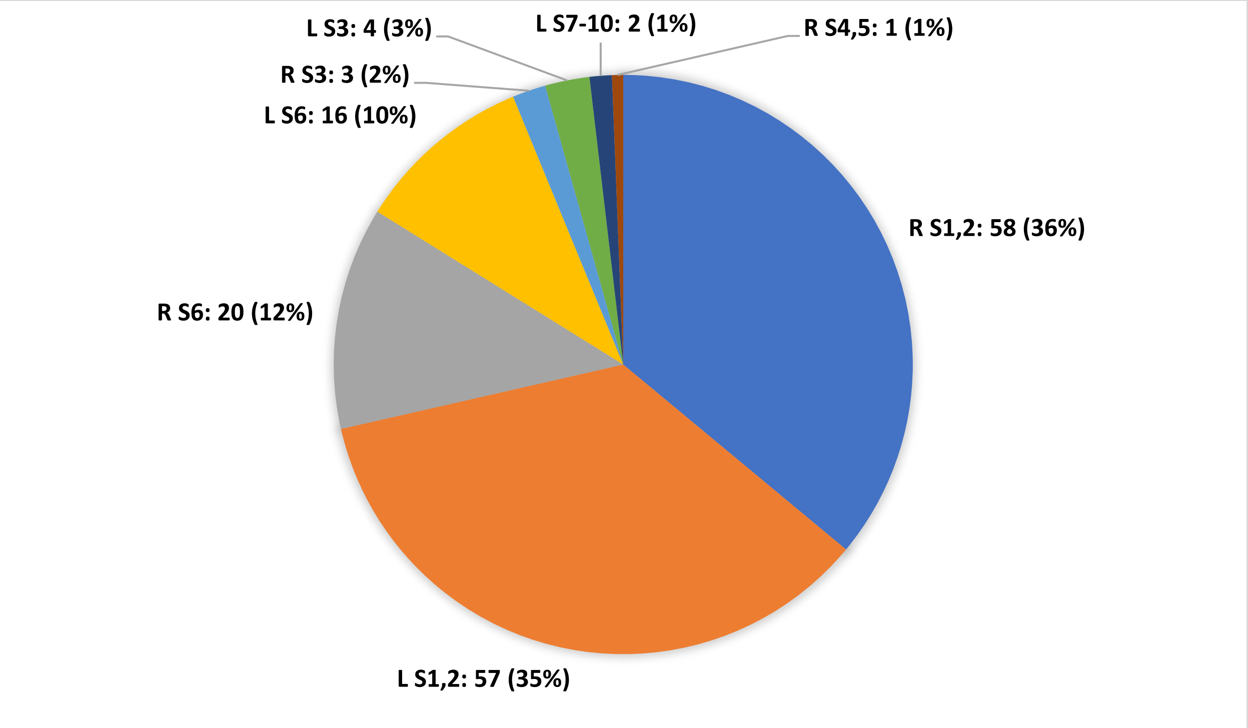

Supplemental Figure 1: Distribution of cavities by baseline pulmonary segment.

Distribution of the number of cavities by pulmonary segment for the 124 participants included in the analysis. The right upper lobe is divided into an apical (S1), posterior (S2), and anterior (S3) segments. The right middle lobe is divided into a lateral (S4) and medial (S5) segments. The left upper lobe is divided into apicoposterior (S1,2), anterior (S3), superior lingular (S4), and inferior lingular (S5) segments. The right lower lobe is divided into superior (S6), medial (S7), anterior (S8), lateral (S9), and posterior (S10) segments. The left lower lobe is divided into superior (S6), anteromedial (S7,8), lateral (S9), and posterior (S10) segments. R=right, L=left.

Supplemental Figure 2

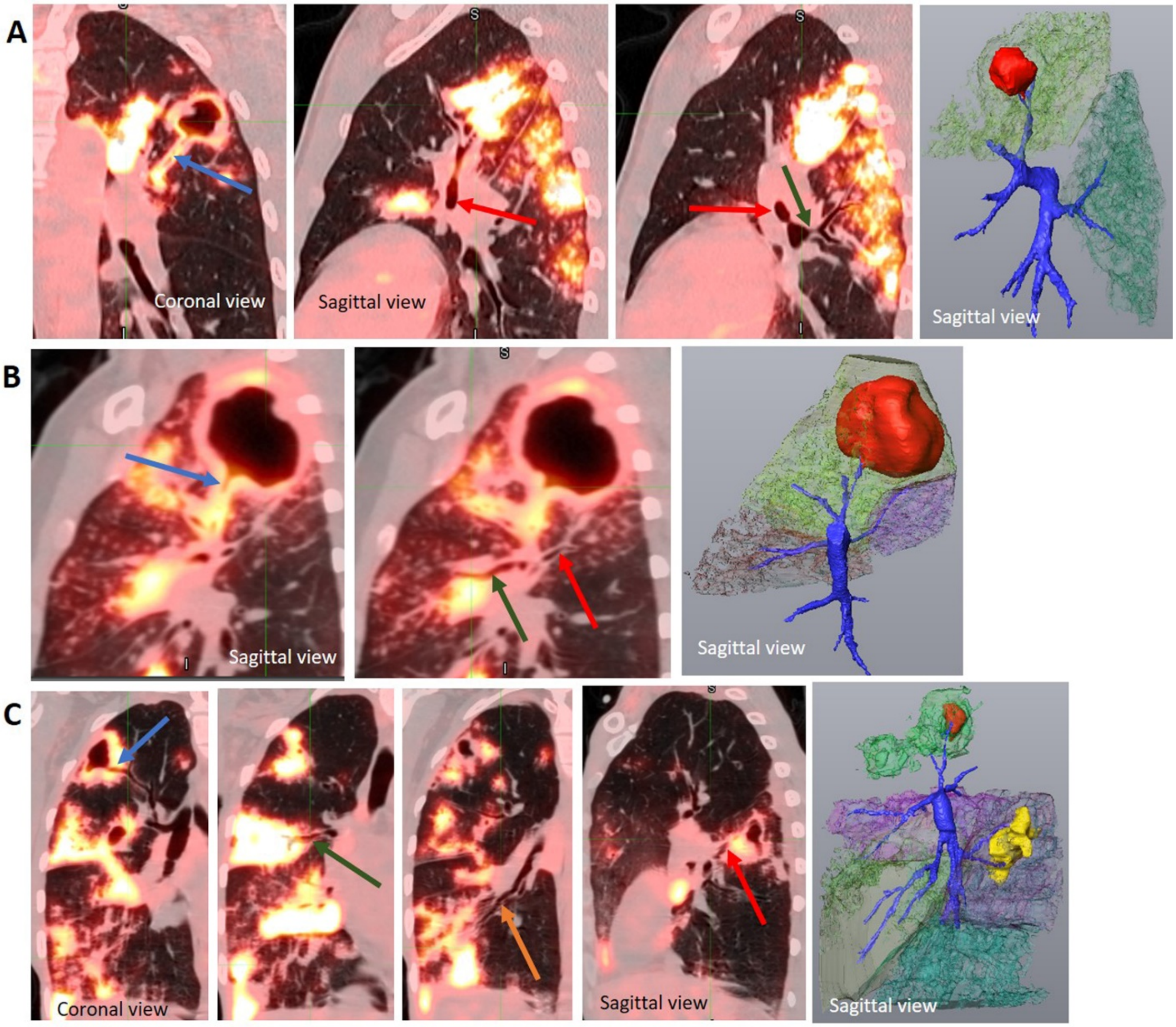

D

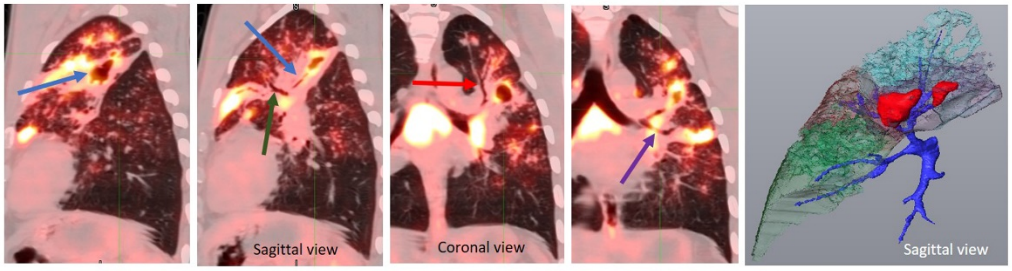
Sagittal view
Coronal view
Sagittal view

E

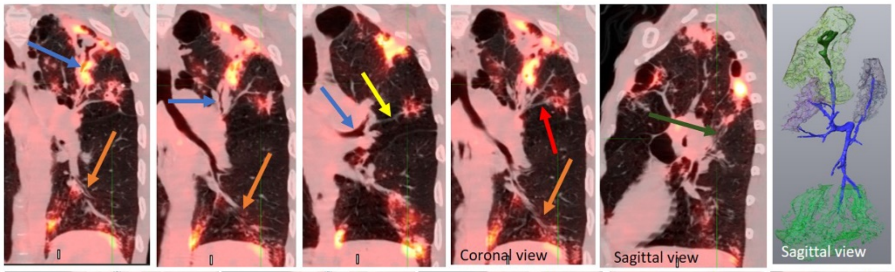
Coronal view
Sagittal view
Sagittal view

F

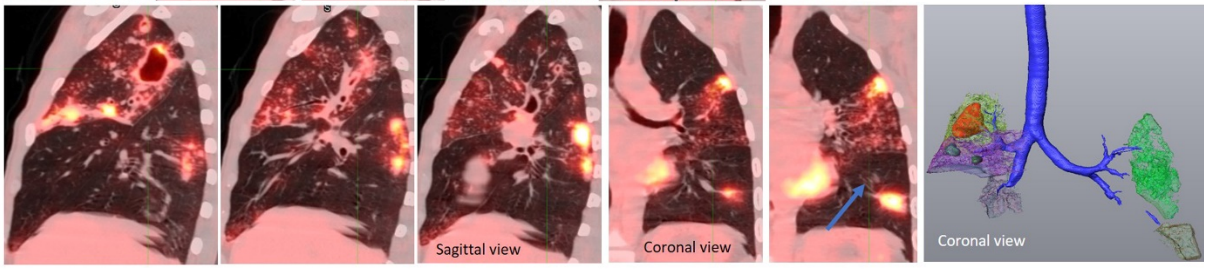
Sagittal view
Coronal view
Coronal view

D

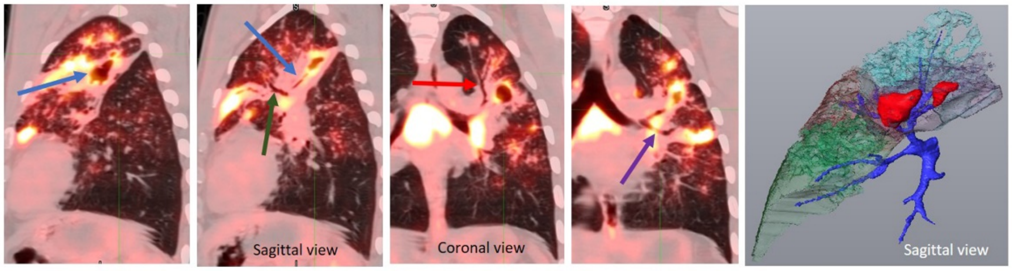
Sagittal view
Coronal view
Sagittal view

E

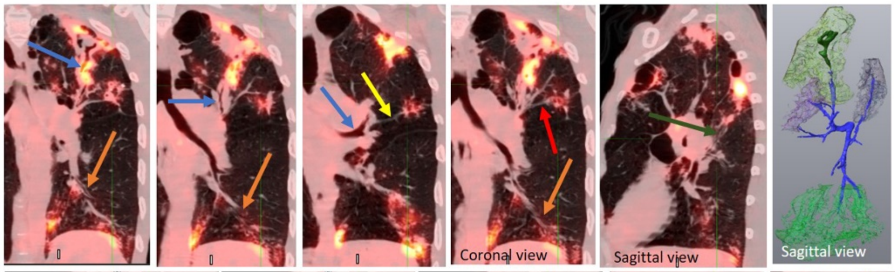
Coronal view
Sagittal view
Sagittal view

F

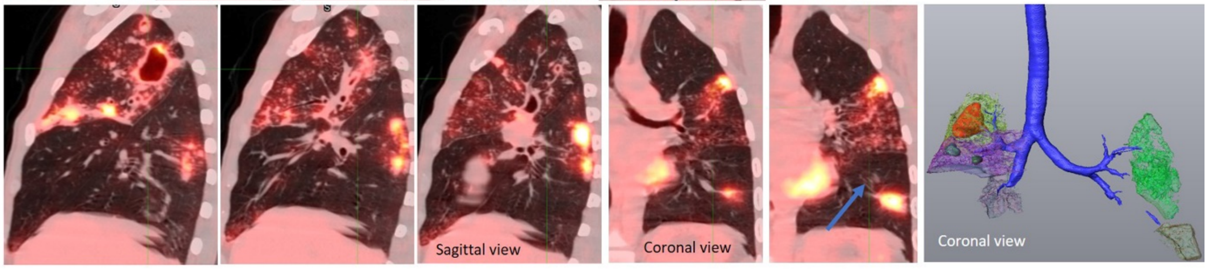
Sagittal view
Coronal view
Coronal view

J

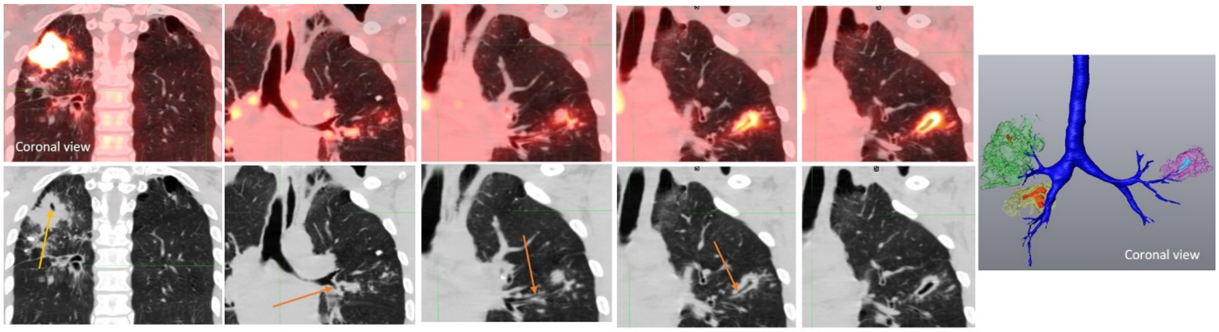

K

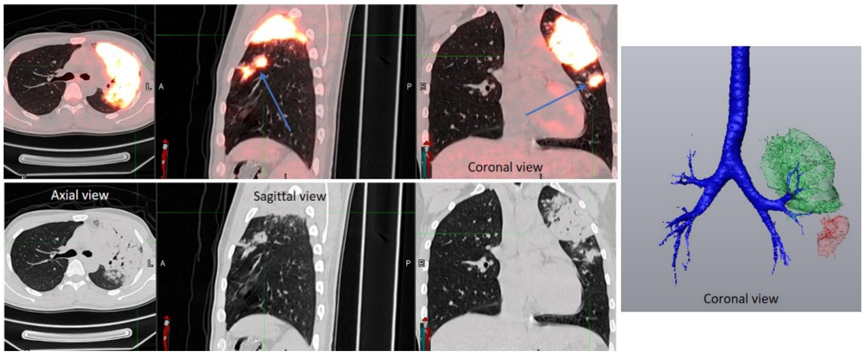

Supplemental Figure 2: Additional examples of tuberculosis bronchial spread patterns on baseline PET-CT scans. (A) Bronchus drains left S2 segment cavity (blue arrow) down left main bronchus (red arrow) to the left S6 segment bronchus (green arrow), causing disease in apex of the left lower lobe. In the 3D rendering, the trachea is removed to allow better visualization. (B) Right apical cavity drains down S1 segment bronchus (blue arrow) to cause disease posteriorly via the S2 segment bronchus (red arrow) and anteriorly via the S3 segment bronchus (green arrow). In the 3D rendering, the trachea is removed to allow better visualization. (C) Right S1 segment cavity with clear bronchial connection (blue arrow) to right main bronchus, with bronchial connection to right S3 segment consolidation (green arrow). There is also clear bronchial connection (orange arrow) to right S8,9 segment consolidation, which could have spread from right S1 cavity or from right S6 cavity, which also connects to right main bronchus (red arrow). In the 3D rendering, the trachea is removed to allow better visualization. (D) Two cavities in left S2 segment with bronchial connections (blue arrows) to left main bronchus, with bronchial connections to S3 segment anterior (green arrow), S1 segment superior (red arrow), and S4,5

lingula (purple arrow). In the 3D rendering, the trachea is removed to allow better visualization. (E) Elongated cavity still containing some caseum in left S1 segment with bronchial connection drains down to left main bronchus (blue arrow). The lesion just inferior has two bronchial connections, a superior one (red arrow) and an inferior one (yellow arrow). The left S6 segment then comes off the left bronchial tree and spreads disease posteriorly to the apex of the left lower lobe (green arrow). Finally, the left bronchial tree ends in the base of the left lower lobe, causing more disease (orange arrows). In the 3D rendering, the trachea is removed to allow better visualization. (F) Right S2 segment has a 12.7 mL cavity and right S3 segment has 2 small <1 mL cavities. Both drain down the right main bronchus to right S6 segment. They also drain across the carina to the left upper lobes S3,4,5. There is also consolidation in left S9 segment. Although a bronchial connection is not clearly visualized all the way to the S9 lesion, there is a small area of inflammation just proximal to S9 where a small area of bronchus can be seen (blue arrow). (G) Right S1 segment cavity drains down the right main bronchus, across the carina, and into the left S3 segment to cause disease. A superior (blue arrow) and inferior (red arrow) bronchial connection to left S3 consolidation is seen. Note that bronchial spread across the carina could result from gravity in a patient who sleeps on his/her side at night. (H) Large left S2 segment cavity and smaller S3 segment cavity drain down left main bronchus to cause disease in the left lingula and lower lobe. (I) Left upper lobe S2 segment cavity drains across the carina into the right upper lobe S2 segment. (J) Dense consolidation in the right upper lobe S2 segment with intense FDG uptake (SUVmax 10.5) and a very small cavity (about 0.15 mL) better seen on the CT scan (yellow arrow). There is a slightly larger cavity (1.75 mL) with mild FDG uptake (SUVmax 2.6) in the apical S6 segment of the right lower lobe. A bronchial pathway can be traced to a lesion in the left upper lobe S3 segment (orange arrows), directly into an inflamed bronchus (light blue on 3D drawing) that possibly is becoming a cavity. (K) Dense left upper lobe consolidation with intense FDG uptake (SUVmax 19.2) involving segments S2 and S3 with air

bronchograms but no visualized cavity. There appears to be bronchial spread to segment S4 (blue arrows; red lesion in 3D drawing) in the lingula.

Supplemental Figure 3

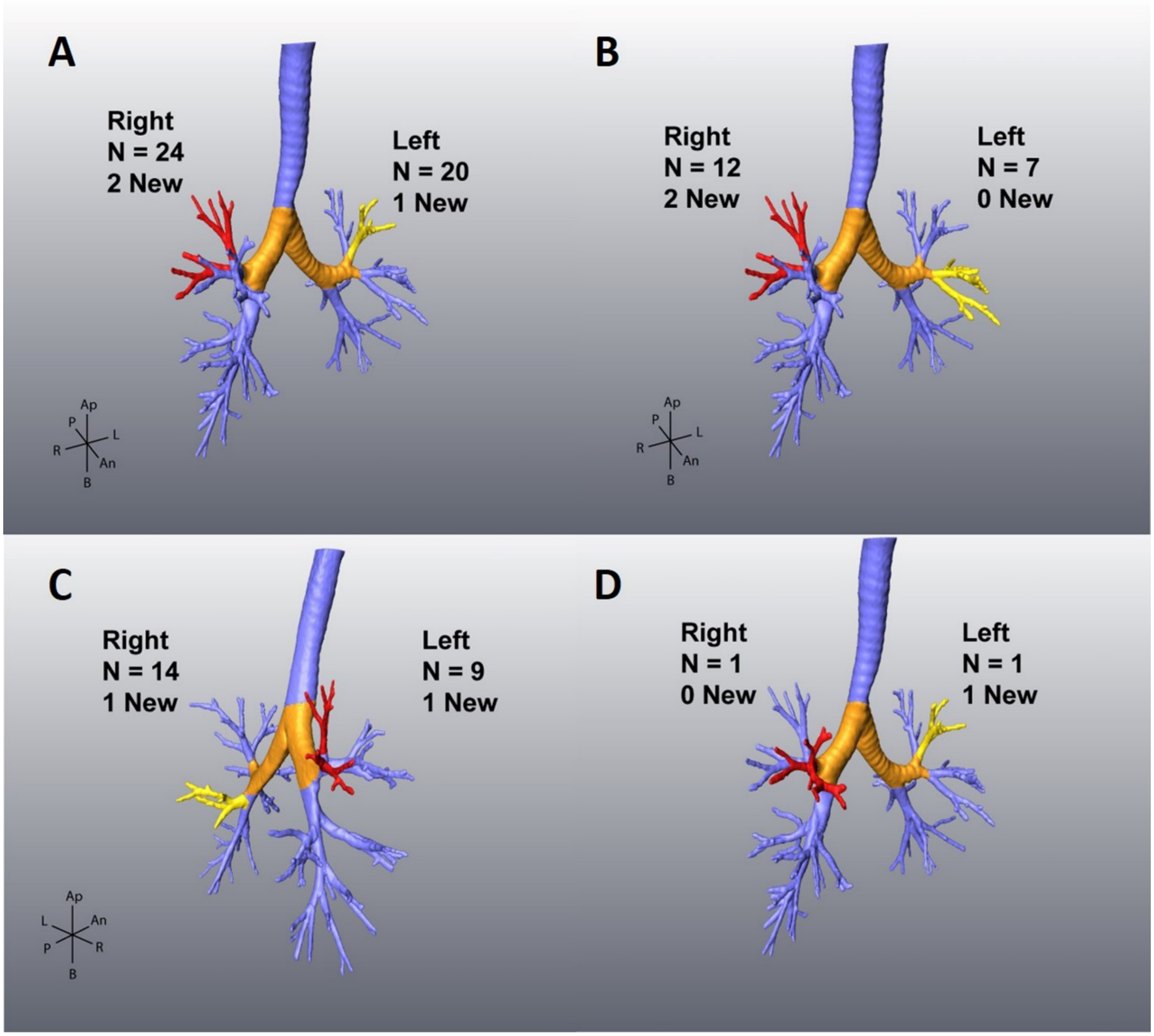

Supplemental Figure 3: Contralateral bronchial spread pathways for apical segments.

Additional 3D renderings of the source (cavitary) pulmonary segment (red), the bronchial spread pathway (orange), and the destination pulmonary segment (yellow) of selected bronchial spread pathways. The numbers indicate the total occurrences (baseline and week 4) of this pattern among the 124 participants analyzed, with the number of new week 4 occurrences (subset of total). Only the right sided source to left sided destination pathways are depicted. (A) Right S1,2 to left S3 and left S1,2 to

right S3. (B) Right S1,2 to left S4,5 and left S1,2 to right S4,5. (C) Right S1,2 to left S6 and left S1,2 to right S6. (D) Right S3 to left S3 and left S3 to right S3.
